# Description of a new species of *Saissetia* from China (Hemiptera, Coccomorpha, Coccidae)

**DOI:** 10.3897/zookeys.791.27186

**Published:** 2018-10-22

**Authors:** Na Zhang, Tong Cao, Ji-Nian Feng

**Affiliations:** 1 Key Laboratory of Plant Protection Resources and Pest Management, Ministry of Education, Entomological Museum, College of Plant Protection, Northwest A&F University, Yangling, Shaanxi Province, 712100, China Northwest A&F University Yangling China

**Keywords:** Coccinae, distribution, *
Saissetia
*, soft scale insect, taxonomy

## Abstract

The adult female of a new species of soft scale *Saissetiapuerensis* Zhang & Feng, **sp. n.** is described and illustrated from the genus *Saissetia* Deplanche, 1859. This species was collected on *Lithocarpusuvariifolius* (Hance) in Yunnan province, China. A key is provided to separate adult females of all *Saissetia* species known from China. A table is provided showing the distribution of *Saissetia* in various zoogeographical regions throughout the world.

## Introduction

Soft scale insects, the third largest family of the Coccoidea, are distributed around the world, and currently include 169 genera and 1183 species (García Morales 2018). Most of them are pests of agricultural and horticultural crops. One species of soft scale, *Ericeruspela* which provides wax, an important industrial raw material, is considered to be beneficial in industry (Fang and Wang 2012; Henderson and Hodgson 2005).

The genus *Saissetia*, described by Deplanche in 1859, is a member of the tribe Saissetiini, subfamily Coccinae, and includes 44 species from around the world (Tang 1991; García Morales 2018). Six species of *Saissetia* have been recorded in China (García Morales 2018).

In this paper, the adult female of a new species *Saissetiapuerensis* Zhang & Feng, sp. n. is described and illustrated. In addition, the genus *Saissetia* is described and a key is provided to separate the six species of *Saissetia* currently known from China. A list of *Saissetia* species throughout the world and their distributions in various zoogeographical regions is presented in Table 1.

## Materials and methods

All specimens were collected from Yunnan province in China, and mounted according to the methods described by Hodgson and Henderson (2000). The morphological terminology describing the mounted specimens primarily follows the nomenclature developed by Hodgson (1994). A Nikon compound microscope was used to examine specimens and an Olympus BH–2 stereoscopic microscope was used to draw illustrations from mounted adult female specimens. Measurements of all characters were recorded in micrometers (μm) or millimeters (mm).

All specimens were deposited in the Northwest A&F University, Yangling, Shaanxi, China (**NWAFU**).

## Taxonomy

### Genus Saissetia

Taxon classificationAnimaliaHemipteraCoccidae

Deplanche, 1859

Saissetia Deplanche, 1859: 6.
Bernardia Ashmead, 1891: 100.

#### Type species.

*Lecaniumcoffeae* Walker, 1852.

#### Generic diagnosis.

**Adult female.** Body oval, slightly or distinctly convex, H-shaped ridge distinctively present on dorsal surface. ***Dorsum*.** Derm membranous, oval or polygonal areolations. Dorsal setae coniform; dorsal tubercles present or absent; dorsal tubular ducts absent; anal plate triangular, with obvious discal seta. ***Margin*.** Marginal setae branched or apex pointed; stigmatic spines of three setae, the median spine longer than others; stigmatic cleft shallow or deep. **Venter.** Antennae of 6–8 segments; legs well developed, with tibio-tarsal articulation sclerosis (except in *S.neglecta*); spiracular disc-pores with 5–6 loculi; pregenital disc-pores with 10–12 loculi, present around anal plate, some on abdominal segment, a few pregenital disc-pores extend to thorax; ventral tubular ducts present in submargin.

### Saissetia puerensis

Taxon classificationAnimaliaHemipteraCoccidae

Zhang & Feng sp. n.

http://zoobank.org/9910C83A-8F63-464E-9181-DEEB352340FF

Figure 1

#### Material examined.

**Holotype**: adult female. CHINA, Yunnan, Puer. 24. vii. 2017, on *Lithocarpusuvariifolius* (Hance) Rehd, Na Zhang (NWAFU). **Paratypes**: two adult females mounted on different slides, data same as holotype.

#### Diagnosis.

The adult female of *S.puerensis* can be diagnosed by the combination of the following features: (1) body convex and sclerotized, distinct H-shaped ridge present on dorsum surface; (2) dorsal tubercles present; (3) dorsal tubular ducts absent; (4) dorsal setae tapered; (5) antennae 8 segments; (6) legs well developed, with tibio-tarsal articulation and articulation sclerosis; (7) spiracular disc-pores present in a rather broad band 7–8 pores wide; (8) anal plates with a discal seta; (9) ano-genital fold with four or five pairs of setae; (10) four types of ventral tubular ducts: (i) type I present on medial submarginal area and inner and medial submarginal area of posterior abdominal segments, some scattered on inner submarginal area mingling with type II, some on outer submarginal area mingling with type III, (ii) type II present mainly on inner submarginal area, few present on procoxa and mesocoxa, and a few ducts present near antennae and mouthparts, some mingling with type I in medial submarginal area, (iii) type III present on outer broad submarginal area, some ducts present in inner submarginal area, (iv) type IV present on anal cleft and broad submarginal band mingling with t types I, II and III, most present on posterior abdominal segments, few ducts present on mesocoxa; (11) pregenital disc-pores, 10–11 loculi, mainly with ten loculi, abundant around anal opening, some extending in transverse bands on abdominal segments, and some laterad of metacoxa and mesocoxa.

**Figure 1.**
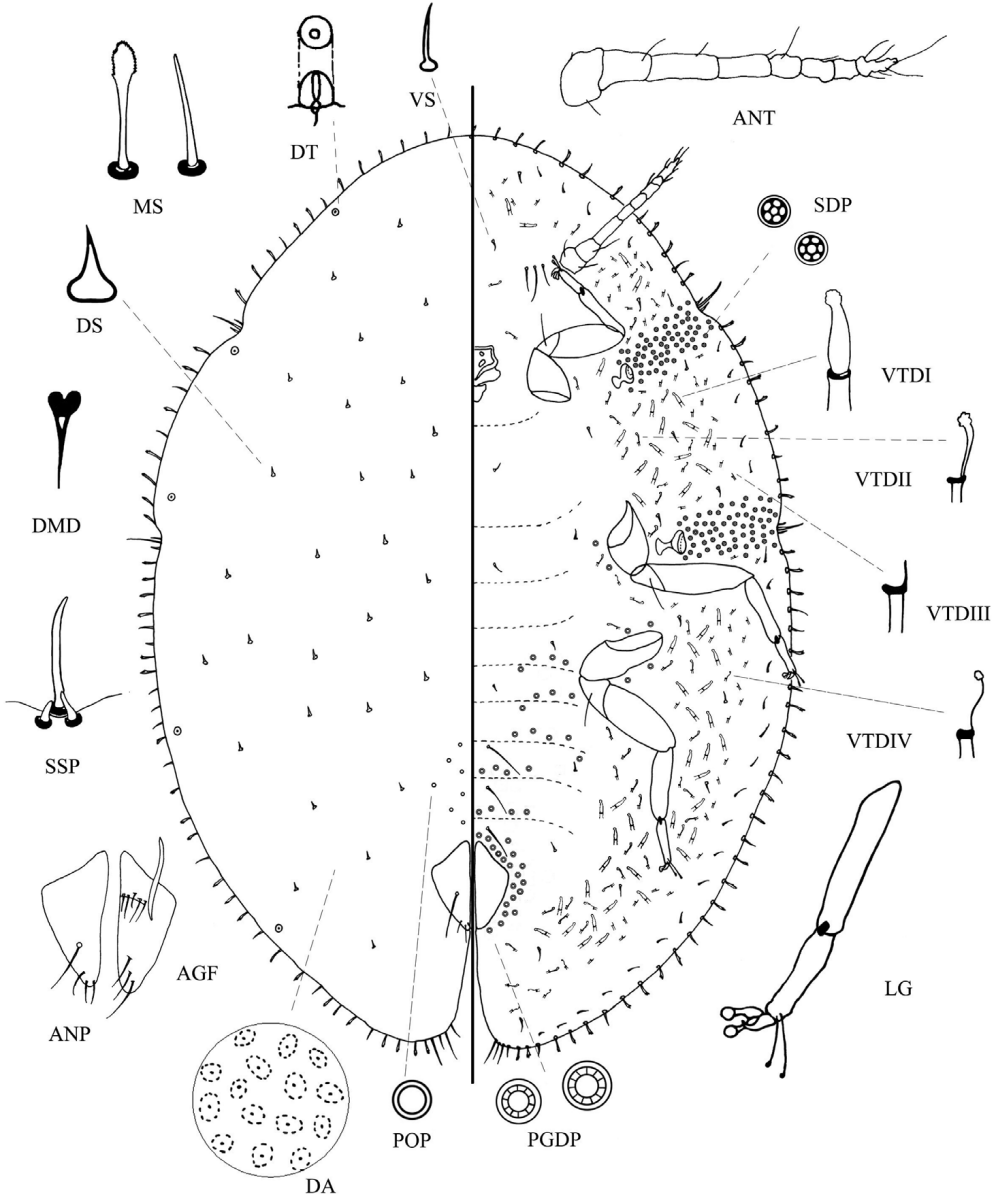
*Saissetiapuerensis* Zhang & Feng, sp. n., adult female. The dorsal surface is depicted on the left side and the ventral surface on the right side, with enlargements of some important characters shown around the main illustration. Abbreviations: **AGF** ano-genital fold **ANP** anal plates **ANT** antenna **DA** dermal areolations **DMD** dorsal microduct **DS** dorsal seta **DT** dorsal tubercles **LG** tibio-tarsus of hind leg **MS** marginal setae **PGDP** pregenital disc-pore **POP** preopercular pores **SDP** spiracle disc-pore **SSP** stigmatic spine **VTD** ventral tubular ducts of types I-IV **VS** ventral setae.

#### Description.

***Appearance in life*.** Pre-reproductive adult female: Body elongate-oval, dorsum greenish. Mature adult female: Body oval, dorsum reddish-brown, convex and sclerotized, distinct H-shaped ridge present on dorsum surface.

***Slide-mounted specimens***: Body oval, 1.5–2.3mm long, 1.0–1.5mm wide, margin with a distinct indentation at each stigmatic cleft; anal cleft 1/8–1/10 body length.

***Dorsum***: Derm membranous or slight sclerotized. Dermal areolations well developed, with one dorsal microductule. Dorsal setae 5.3–7.8 μm long, tapered, apex slightly curved, with a well-developed basal socket, scattered all over the dorsum. Dorsal tubercles present on submarginal area, 2–5 on head, 0–2 between stigmatic clefts, 2–3 between posterior stigmatic cleft and anal cleft. Preopercular pores small, 2.7–4.5 μm wide, 14–26 in front of anal plates. Dorsal tubular ducts absent. Anal plates together quadrate, 139.6–155.1 μm long, 67.5–78.1 μm wide, outer angle slightly obtuse; posterolateral margin slightly longer than anterolateral: anterolateral margin 84.5–100.9 μm long, posterolateral margin 103.5–122.3 μm long; posterolateral margin slightly convex and anterolateral margin slightly concave, with four apical setae and a discal seta; supporting bars not contacted with each other. Ano-genital fold with four or five pairs of setae, 37.1–60.7 μm long, present along anterior margin; three pairs of setae, 28.6–57.2 μm long, present along lateral margin. Anal ring subcircular, with four pairs of setae, 163.1–189.4 μm long. Eyespots near margin.

***Margin***: Marginal setae, 12.8–44.1 μm long, branched, straight or curved, a few spinous, all with well-developed basal sockets, with 36–46 setae between anterior clefts, 12–16 setae between each anterior cleft and posterior cleft, and 27–35 setae between each posterior cleft and anal cleft; some over 50 μm around anal cleft. Stigmatic cleft distinct and deep, with three stigmatic spines: one medial spine, 60.3–65.9 μm long, blunt, slightly curved apex and broadly based; lateral spines, 15.5–21.2 μm long, stout, straight; median four to five times longer than laterals.

***Venter***: Derm membranous. Antennae 8 segments, 328.6–357.2 μm long, the third segment longest; with three pairs of interantennal setae, 21.4–88.5 μm long. Spiracular disc-pores, with 5–6 loculi, mainly with five loculi, occasionally six loculi, each about 3.2–4.9 μm wide; present in a rather broad band 7–8 pores wide between stigmatic cleft and each spiracle; 30–66 pores in each anterior spiracle band and 45–93 pores in each posterior band. Legs well developed, slender, with tibio-tarsal articulation and articulation sclerosis; claws without denticle; tarsal digitules and claw digitules both with knobbed apices, but tarsal digitules longer and thinner than claw digitules, tarsal digitules length 49.5–66.7 μm, claw digitules length 27.2–36.0 μm; dimensions of metathoracic leg: coxa 105.2–135.4 μm, trochanter+ femur 182.3–230.3 μm, tibia + tarsus 229.6–249.8 μm, tibia about two times longer than tarsus, claw 21.4–27.9 μm. Pregenital disc-pores, 10–11 loculi, mainly with ten loculi, occasionally 11 loculi, each about 3.8–5.9 μm wide; abundant around anal opening, some extending in transverse bands on abdominal segments, and some laterad of metacoxa and mesocoxa; with three pairs of long pregenital setae, each 95.7–138.3 μm long. There are four types of ventral tubular ducts:

Type I: a duct with large terminal gland, inner ductule almost as wide and long as outer ductule; present on medial submarginal area and inner and medial submarginal area of posterior abdominal segments, some scattered on inner submarginal area mingling with type II, some on outer submarginal area mingling with type III.

Type II: inner ductule almost twice as long as outer ductule, inner ductule thinner than outer ductule, but not filamentous, with a well-developed terminal gland; present mainly on inner submarginal area, few present on procoxa and mesocoxa, and a few ducts present near antennae and mouthparts, some mingling with type I in medial submarginal area.

Type III: outer ductule of this type slightly shorter than type I, a filamentous inner ductule without terminal gland; present on outer broad submarginal area, some ducts present in inner submarginal area.

Type IV: inner ductule almost two times as long as outer ductule, a filamentous inner ductule with a ball-shaped terminal gland; present on anal cleft and broad submarginal band mingling with t types I, II, and III, most present on posterior abdominal segments, few ducts present on mesocoxa. Ventral tubular ducts distributed irregularly; a few are scattered near anal cleft, becoming progressively more frequent anteriorly. Submarginal setae present in a single row, each 7.5–14.2 μm long; other ventral setae slender, each 7.9–18.6 μm long, quite sparsely distributed.

#### Etymology.

The species epithet *puerensis* is a noun in apposition, referring to the place where this new species was collected.

#### Host.

*Lithocarpusuvariifolius* (Hance) Rehd in China.

#### Distribution.

China (Yunnan).

##### Key to adult females of *Saissetia* occurring in China

**Table:** 

1	Stigmatic spines 4–7 setae	***S.vivipara* Williams &Watson, 1990**
–	Stigmatic spines 3 setae	**2**
2	Ventral tubular ducts with a broad inner ductule present in submarginal area	**3**
–	Ventral tubular ducts with a broad inner ductule absent in submarginal area	**5**
3	Marginal setae fine; dorsal submarginal tubercles absent	***S.bobuae* Takahashi, 1935**
–	Marginal setae branched; dorsal submarginal tubercles present	**4**
4	Ventral tubular ducts of 3 types distributed regularly in a submarginal band (*type I* distributed on medial submarginal area, *type II* distributed on inner submarginal area and *type III* distributed on outer submarginal area); Spiracular band 2–3 pores wide	***S.coffeae* (Walker), 1852**
–	Ventral tubular ducts of 4 types distributed irregularly in a submarginal band (the distribution of *types I*, *II*, *III and IV* is irregular, *type I* mingling with *type II* on inner submarginal area and mingling with *type III* on outer submarginal area); Spiracular band 7–8 pores wide	***S.puerensis* sp. n.**
5	Marginal setae between each anterior cleft and posterior cleft number 15–23	***S.miranda* (Cockerell & Parrott), 1899**
–	Marginal setae between each anterior cleft and posterior cleft number 4–12	**6**
6	Marginal setae branched; anal ring with four pairs of setae	***S.neglecta* De Lotto, 1969**
–	Marginal setae fine; anal ring with three pairs of setae	***S.oleae* (Olivier), 1791**

## Discussion

This species is considered to be close to *Saissetiacoffeae* (Choi and Lee 2017) and they share some distinct characteristics: 1) more than one type of ventral tubular duct; 2) anal plate with a discal seta; 3) three pairs of setae present along lateral margin; and 4) anal ring subcircular, with four pairs of setae.

However, *S.puerensis* can be distinguished by the possession of the following features (character states of *S.coffeae* in parenthesis): 1) four types of ventral tubular ducts (three); 2) ventral tubular ducts distributed irregularly, especially on posterior abdominal segments, type I mingling with type II (regularly); 3) type II not present on medial thorax (present); 4) inner ductule of type II ventral tubular ducts almost twice as long as outer ductule (inner ductule as long as outer ductule); 5) spiracle in a rather broad band 7–8 pores wide (2–3); 6) preopercular pores 14–26 in front of anal plates (5–14); and 7) ano-genital fold with four or five pairs of setae (three).

*Lithocarpusuvariifolius* (Hance) Rehd is the only plant known to be a host for *S.puerensis*. Heavy infestations of this pest cause a sooty mold to build up, which reduces photosynthesis and stunts the growth of the plant. *L.uvariifolius* is only known from China (Wu et al. 1999), and *S.puerensis* may therefore be restricted to this country. Further studies are required to determine if *S.puerensis* has other host plants and occurs in other countries.

## World distribution of *Saissetia* species (Table 1)

Table 1 is based on information from ScaleNet which has not been published previously. Only *S.coffeae*, *S.miranda*, and *S.oleae* have worldwide distributions. The highest numbers of species are found in the Ethiopian and Neotropical regions, with 50.0% and 43.2% respectively; 40.9% of species occur only in the Ethiopian region and 34.1% only in the Neotropical region. The Nearctic region has fewest species, with only 9.1%.

**Table 1.** *Saissetia* species of the world: a simple list with indications of distribution by zoogeographical regions. Abbreviations: Pa = Palaearctic, Na = Nearctic, Et = Ethiopian, Or = Oriental, Au = Australian and Oceanic, Nt = Neotropical.

Species	Et	Nt	Or	Au	Pa	Na
* S. absona *	+					
* S. anonae *		+				
* S. auriculata *		+				
* S. bobuae *			+			
* S. carnosa *	+					
* S. cassiniae *				+		
* S. cerei *					+	
* S. chimanimanae *	+					
* S. chitonoides *	+					
* S. coffeae *	+	+	+	+	+	+
* S. discoides *		+				
* S. dura *		+				
* S. ficinum *					+	
* S. glanulosa *		+				
* S. hurae *		+				
* S. infrequens *		+				
* S. jocunda *	+					
* S. lucida *		+				
* S. malagassa *	+					
* S. minensis *		+				
* S. miranda *	+	+	+	+	+	+
* S. mirifica *				+		
* S. monotes *	+					
* S. munroi *	+					
* S. neglecta *		+	+	+		+
* S. nigrella *	+					
* S. oleae *	+	+	+	+	+	+
* S. opulenta *	+					
* S. orbiculata *	+					
* S. persimilis *	+					
* S. poinsettiae *	+					
* S. privigna *	+		+		+	
* S. reticulata *		+				
* S. sclerotica *	+					
* S. scutata *		+				
* S. socialis *		+				
* S. somereni *	+					
* S. subpatelliformis *	+					
* S. tolucana *		+				
* S. velfozoi *		+				
* S. vivipara *			+	+		
* S. xerophila *	+					
* S. zanthoxylum *		+				
* S. zanzibarensis *	+					

## Supplementary Material

XML Treatment for Saissetia

XML Treatment for Saissetia puerensis
